# Distinct patterns of copy number alterations may predict poor outcome in central nervous system germ cell tumors

**DOI:** 10.1038/s41598-023-42842-3

**Published:** 2023-09-21

**Authors:** Hirokazu Takami, Kaishi Satomi, Kohei Fukuoka, Taishi Nakamura, Shota Tanaka, Akitake Mukasa, Nobuhito Saito, Tomonari Suzuki, Takaaki Yanagisawa, Kazuhiko Sugiyama, Masayuki Kanamori, Toshihiro Kumabe, Teiji Tominaga, Kaoru Tamura, Taketoshi Maehara, Masahiro Nonaka, Akio Asai, Kiyotaka Yokogami, Hideo Takeshima, Toshihiko Iuchi, Keiichi Kobayashi, Koji Yoshimoto, Keiichi Sakai, Yoichi Nakazato, Masao Matsutani, Motoo Nagane, Ryo Nishikawa, Koichi Ichimura

**Affiliations:** 1 Department of Neurosurgery, Faculty of Medicine, The University of Tokyo, 7-3-1, Hongo, Bunkyo-ku, Tokyo, 113-8655 Japan; 2 Department of Pathology, Kyorin University Faculty of Medicine, 6-20-2, Shinkawa, Mitaka City, Tokyo, 181-8611 Japan; 3 Departments of Hematology/Oncology, Saitama Children’s Medical Center, 1-2, Shintoshin, Chuo-ku, Saitama City, Saitama 330-8777 Japan; 4 Department of Neurosurgery, Graduate School of Medicine, Yokohama City University, 3-9, Fukuura, Kanazawa-ku, Yokohama City, Kanagawa 236-0004 Japan; 5 Department of Neurosurgery, Graduate School of Medical Sciences, Kumamoto University, 1-1-1, Honjo, Chuo-ku, Kumamoto, 860-8556 Japan; 6 Department of Neuro-Oncology/Neurosurgery, Saitama Medical University International Medical Center, 1397-1, Yamane, Hidaka City, Saitama 350-1298 Japan; 7 Department of Neurosurgery, Jikei University, 3-25-8, Nishi-shinbashi, Minato-ku, Tokyo, 105-8461 Japan; 8 Department of Clinical Oncology and Neuro-Oncology Program, Faculty of Medicine, Hiroshima University, 1-2-3, Kasumi, Minami-ku, Hiroshima, 734-8551 Japan; 9 Department of Neurosurgery, Tohoku University School of Medicine, 1-1, Seiryo-machi, Aoba-ku, Sendai City, Miyagi 980-8574 Japan; 10 Department of Neurosurgery, Kitasato University, 1-15-1 Kitasato, Minami, Sagamihara, Kanagawa 252-0374 Japan; 11 Department of Neurosurgery, Graduate School of Medical and Dental Sciences, Tokyo Medical and Dental University, 1-5-45, Yushima, Bunkyo-ku, Tokyo, 113-0034 Japan; 12 Department of Neurosurgery, Kansai Medical University Hospital, 2-3-1, Shinmachi, Hirakata City, Osaka 573-1191 Japan; 13 Department of Neurosurgery, University of Miyazaki Faculty of Medicine, 5200, Kihara, Kiyotakecho, Miyazaki, 889-1692 Japan; 14 Department of Neurosurgery, Chiba Cancer Center, 666-2, Nitona-cho, Chuo-ku, Chiba, 260-0801 Japan; 15 Department of Neurosurgery, Kyorin University Faculty of Medicine, 6-20-2, Shinkawa, Mitaka City, Tokyo, 181-8611 Japan; 16 Department of Neurosurgery, Kyusyu University Hospital, 3-1-1, Maidashi, Higashi-ku, Fukuoka, 812-8582 Japan; 17 Shinshu Ueda Medical Center, 1-27-21, Midorigaoka, Ueda City, Nagano, 386-8610 Japan; 18 Department of Pathology, Hidaka Hospital, 886, Nakaomachi, Takasaki City, Gunma 370-0001 Japan; 19 Gotanda Rehabilitation Hospital, 8-20, Nishi-gotanda, Shinagawa-ku, Tokyo, 141-0031 Japan; 20 Department of Brain Disease Translational Research, Juntendo University Graduate School of Medicine, 2-1-1 Hongo, Bunkyo-ku, Tokyo, 113-8421 Japan

**Keywords:** Medical research, Neurology, Risk factors, Signs and symptoms, Neurological disorders

## Abstract

We have previously reported that 12p gain may predict the presence of malignant components and poor prognosis for CNS germ cell tumor (GCT). Recently, 3p25.3 gain was identified as an independent predictor of poor prognosis for testicular GCT. Eighty-one CNS GCTs were analyzed. Copy number was calculated using methylation arrays. Five cases (6.2%) showed 3p25.3 gain, but only among the 40 non-germinomatous GCTs (NGGCTs) (5/40, 12.5%; *p* = 0.03). Among NGGCTs, those with a yolk sac tumor component showed a significantly higher frequency of 3p25.3 gain (18.2%) than those without (1.5%; *p* = 0.048). NGGCTs with gain showed significantly shorter progression-free survival (PFS) than those without (*p* = 0.047). The 3p25.3 gain and 12p gain were independent from each other. The combination of 3p25.3 gain and/or 12p gain was more frequent among NGGCTs with malignant components (69%) than among those without (29%; *p* = 0.02). Germinomas containing a higher number of copy number alterations showed shorter PFS than those with fewer (*p* = 0.03). Taken together, a finding of 3p25.3 gain may be a copy number alteration specific to NGGCTs and in combination with 12p gain could serve as a marker of negative prognosis or treatment resistance. Germinoma with frequent chromosomal instability may constitute an unfavorable subgroup.

## Introduction

Germ cell tumors (GCTs) occur in various organs in the body, mainly in the gonads (testis and ovary), but also in midline structures such as brain, mediastinum and sacrococcygeal regions^1^. Extragonadal GCT is hypothesized to develop from a mis-migrated primordial germ cell (PGC), which is pluri- or toti-potent^2^. Central nervous system (CNS) GCTs are mostly categorized as type II when classified according to the seven types of developmental potential. Type II GCTs comprise CNS GCTs and testicular and ovarian GCTs^1^. The transcriptome and methylation studies comparing CNS and testicular GCTs (TGCTs) have revealed close similarities between germinoma and seminoma, as well as non-germinomatous GCTs (NGGCTs) and non-seminomatous GCTs (NSGCTs), with some diversity between NGGCT and NSGCT in the expression profiles^3^. Copy number alterations appear more pronounced in TGCTs than in CNS GCTs, although the overall profiles show much in common. Thus, these molecular findings corroborate the concept that gonadal and extra-gonadal GCTs are biologically akin to each other. However, the impact of biological findings on clinical phenotypes leaves much to be clarified.

CNS GCTs are treated with platinum-based chemotherapy and radiation therapy, with surgical resection reserved for mature teratoma, unresponsive or remnant tissue after chemotherapy and/or radiation therapy, or biopsy for diagnostic purposes^4–9^. With the exception of some modifications in chemotherapy intensity, radiation dose and coverage in view of the tumor response to initial treatment, treatment regimens are basically homogeneous without stratification into each germinoma and NGGCT subgroup^10–14^. We have previously reported that 12p gain may predict the presence of malignant components and poor prognosis in CNS GCTs^15^. High tumor cell content and atypical location in germinoma cases, and 12p gain in NGGCT cases have been demonstrated as candidate factors for poor prognosis. The identification of age < 6 years old and exceptionally high tumor marker level (AFP > 1000 ng/ml) as poor prognostic factors was an important advance^4^. High tumor cell content and atypical location have also been demonstrated as poor prognostic factors in germinoma cases^8,15–17^. However, treatment is still generally homogeneous except for distinguishing germinoma or NGGCTs, so further investigations are necessary to identify biomarkers for risk stratification.

A recent study demonstrated that 3p25.3 copy number gain had histological and clinical significance for testicular and mediastinal GCTs in male patients^18^. In that study, 3p25.3 copy number gain calculated based on methylation array was associated with cisplatin resistance in GCT cell lines, non-seminomatous histology (particularly yolk sac tumor), and significantly poorer progression-free survival (PFS) and overall survival (OS). The authors suggested that 3p25.3 copy number status could be incorporated into risk classifications^18^.

In light of these findings, we revisited copy number alterations in CNS GCTs and explored their impact on histological malignancy and prognosis. Here, using a cohort of 81 CNS GCT cases with various histological subtypes, we investigated the histological and clinical significance of 3p25.3 copy number profile with the aim of validating this potential risk predictor. We further extended the study to investigate the importance of total numbers of gains and losses genome-wide to assess the prognostic impact on CNS GCTs.

## Materials and methods

### Patients and histopathological diagnoses

A total of 82 CNS GCTs from 11 institutions underwent central histopathological review by an expert neuropathologist (YN) and were diagnosed as primary (not metastatic) CNS GCTs. Among these, tissues from 16 cases were obtained at recurrence. The cohort was identical to that in a previously published study^15^. Clinical information such as age, sex, tumor location, treatment and follow-up data were as previously published^15^. Histopathological breakdown was as follows: 42 germinomas; 22 mixed GCTs; 7 mature teratomas (MTs); 4 immature teratomas (ImTs); 5 yolk sac tumors (YSTs); one embryonal carcinoma (EC); and one choriocarcinoma (CC). With some variability between institutions, treatment regimens were generally based on histopathological diagnoses classifying patients into three risk groups: germinoma group; intermediate prognosis group; and poor prognosis group^7^. Except for MT cases, all cases received platinum-based chemotherapy and radiation therapy at different intensities, according to the above three risk groups. The intermediate prognosis group included ImTs, teratomas with somatic malignancy, and mixed tumors composed mainly of germinoma or teratoma. The poor prognosis group included so-called malignant GCTs (namely CC, YST and EC) and mixed tumors mainly composed of these histological components. Chemotherapy and radiation dose and coverage have been described previously^8^.

This study was approved by the ethics committee at Juntendo University, Tokyo, Japan (Approval No. M20-0295, informed consent was waived from study participants) and the respective local institutional review boards. All experiments were performed in accordance with relevant guidelines and regulations.

### Molecular data

DNA methylation data obtained using Illumina Infinium Human Methylation450 BeadChips (450 K, Illumina, San Diego, CA, USA) for all 82 cases^19^, as well as somatic mutation data obtained by whole-exome or target sequencing for 81 of the 82 cases were used in the current analyses^20^. One case (GCT83, germinoma) was omitted from further analyses, because the quality of the sample was deemed low, considering that the average detection p value of all the probes across chromosomes calculated using the package minfi (version 1.44.0) was greater than 0.001. Copy number calculation was carried out using the conumee package run in the R statistical environment (version 4.0.2), as described previously^15^, with modifications in the steps of raw data preprocessing and combining probes into bins to align with the preceding study in the testicular GCTs^18^. Methylation array probes in the region of 3p25.3 (chr3:8,700,001–11,800,000, HG19) were aggregated into 25 bins (specified as such with at least 25 probes, otherwise settings were default). Regarding the definition of copy number gain, cases with any segment that showed a log2ratio higher than 0.1 within this region (3p25.3; chr3:8,700,001–11,800,000, HG19) were counted as positive for gain, according to the aforementioned publication^18^. The data regarding 12p status were as published^15^. The data regarding mutational status were as published^20^.

The number of segments in which the average value of log2ratio CN was higher than 0.1 (gain) or lower than − 0.1 (loss) was counted in each case.

### Statistical analysis

Non-parametric values were compared using Wilcoxon’s test. Categorical data were compared between subgroups using Fisher’s exact test. Survival data were analyzed using the log-rank test and the results are shown as Kaplan–Meier curves. Cox proportional hazard models were used for multivariate survival analysis. All statistical analyses were carried out using JMP® 16 (SAS Institute Inc., Cary, NC, USA). Values of *p* < 0.05 were considered statistically significant.

### Ethics statement

This study was approved by the ethics committee at Juntendo University, Tokyo, Japan (Approval No. M20-0295) and the respective local institutional review boards.

### Informed consent

Patient consent was waived due to this study being minimal-risk retrospective review.

## Results

### Copy number status and histological classification

**Figure 1:**
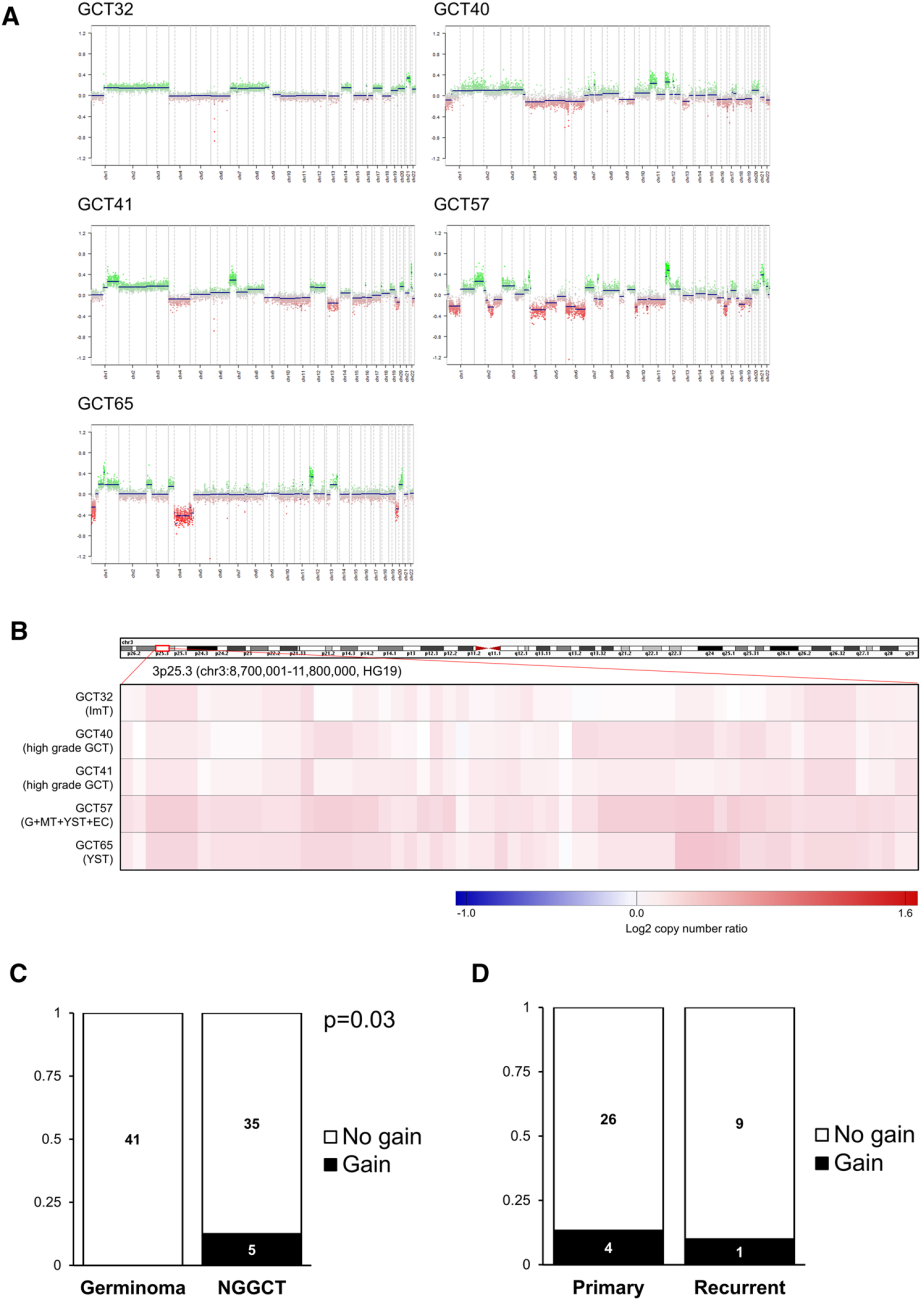
(**A**) Copy number plots for 5 cases with 3p25.3 gain. The 4 cases other than GCT65 showed 3p chromosomal arm gains. (**B**) Heatmap of copy number status within the region of 3p25.3 for the 5 cases acknowledged as harboring a gain. (**C**) Distribution of 3p25.3 gain by histopathological classification. A significantly higher prevalence of 3p25.3 gain was observed in NGGCT cases compared with germinoma cases (*p* = 0.03). (**D**) Distribution of 3p25.3 gain according to primary or recurrent status of the tissues. No difference was observed between groups.

A copy number gain for chromosome 3p25.3 was identified in 5 of the 81 cases (6.2%) (Fig. 1A,B). With the exception of one case (GCT65: YST), the other 4 cases showed 3p whole-chromosomal arm gain, as shown in Fig. 1. Histopathological diagnoses for these five cases were: one YST; one mixed GCT (germinoma + MT + YST + EC); two high-grade GCTs; and one ImT. Gain was observed exclusively in NGGCTs (12.5%), not in germinomas (0%; *p* = 0.03) (Fig. 1C). Correlating with histopathological components, the presence of a YST component was positively associated with the presence of gain (2/11 cases with YST component vs 1/69 cases without YST component; *p* = 0.049). The remaining two cases were high-grade GCTs without further histological specifications. No correlation was observed for any other histopathological component. While the gain was more prevalent among recurrent GCTs in testicular cases, no such correlation was found in CNS NGGCTs (4/30 primary tumors vs. 1/10 recurrent tumors) (Fig. 1D).

### Correlation with mutation profile and 12p gain

The relationship between the presence of mutation in either of the MAPK or PI3K pathways and the presence of copy number gain was investigated in 39 NGGCT cases (mutation information was not available for one case). Mutation in the above two pathways was found in 11 and 4 of the 39 cases, respectively. The 3p25.3 gain was almost evenly distributed, regardless of mutation status (1/11 vs. 4/28, *p* = 1.0 in the MAPK pathway; 1/4 vs. 4/35, *p* = 0.44 in the PI3K pathway). Of note, 3p25.3 gain was mutually exclusive with *KIT* mutation, with 3p25.3 gain observed only in *KIT*-wildtype cases (5/35 cases) and not in any *KIT*-mutant cases (0/4 cases), although the frequency of the two genetic events was not significantly correlated, mainly due to the sample size.

In our previous study, 12p gain was identified as a marker for NGGCT histology^15^. No association was seen between 3p25.3 gain and 12p gain among NGGCT cases (3p25.3 gain: 2/20 12p-neutral cases vs 3/20 12p-gain cases; *p* = 1.0). Combining 12p gain and 3p25.3 gain, the prevalence of these abnormalities among NGGCT cases was 55% (22/40), and the association with NGGCT was more robust (*p* < 0.0001) than that for 12p gain alone (*p* = 0.0002). These abnormalities were found at higher frequency among NGGCT cases with malignant components (18/26, 69%) than among those without (4/14, 29%; *p* = 0.02) (Supplementary Fig. 1).

### Prognostic significance of 3p25.3 gain

**Figure 2:**
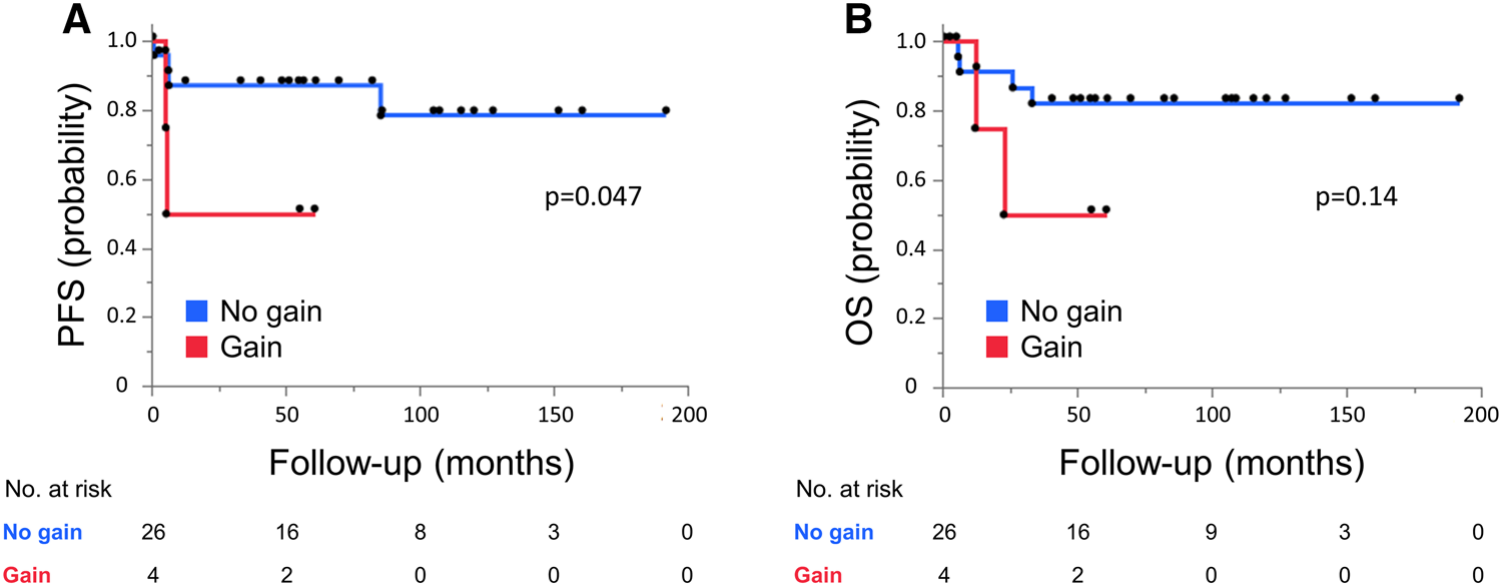
(**A**) Progression-free survival (PFS) among 30 cases of primary (not recurrent) non-germinomatous GCTs was compared between cases with and without 3p25.3 gain. Those with 3p25.3 gain showed significantly shorter PFS than those without (*p* = 0.047). (**B**) Comparison of overall survival (OS) among 30 cases of primary (not recurrent) non-germinomatous GCTs between cases with and without 3p25.3 gain. No significant difference was found, although a trend was seen toward shorter OS in cases with 3p25.3 gain.

Survival analyses were performed for 30 primary NGGCT cases. PFS was significantly shorter in cases with 3p25.3 gain (*p* = 0.047) (Fig. 2A). Cases with 3p25.3 gain tended to have a shorter OS compared with cases without 3p25.3 gain, but the difference was not significant (*p* = 0.14) (Fig. 2B).

### Genome-wide chromosomal aberrations

**Figure 3:**
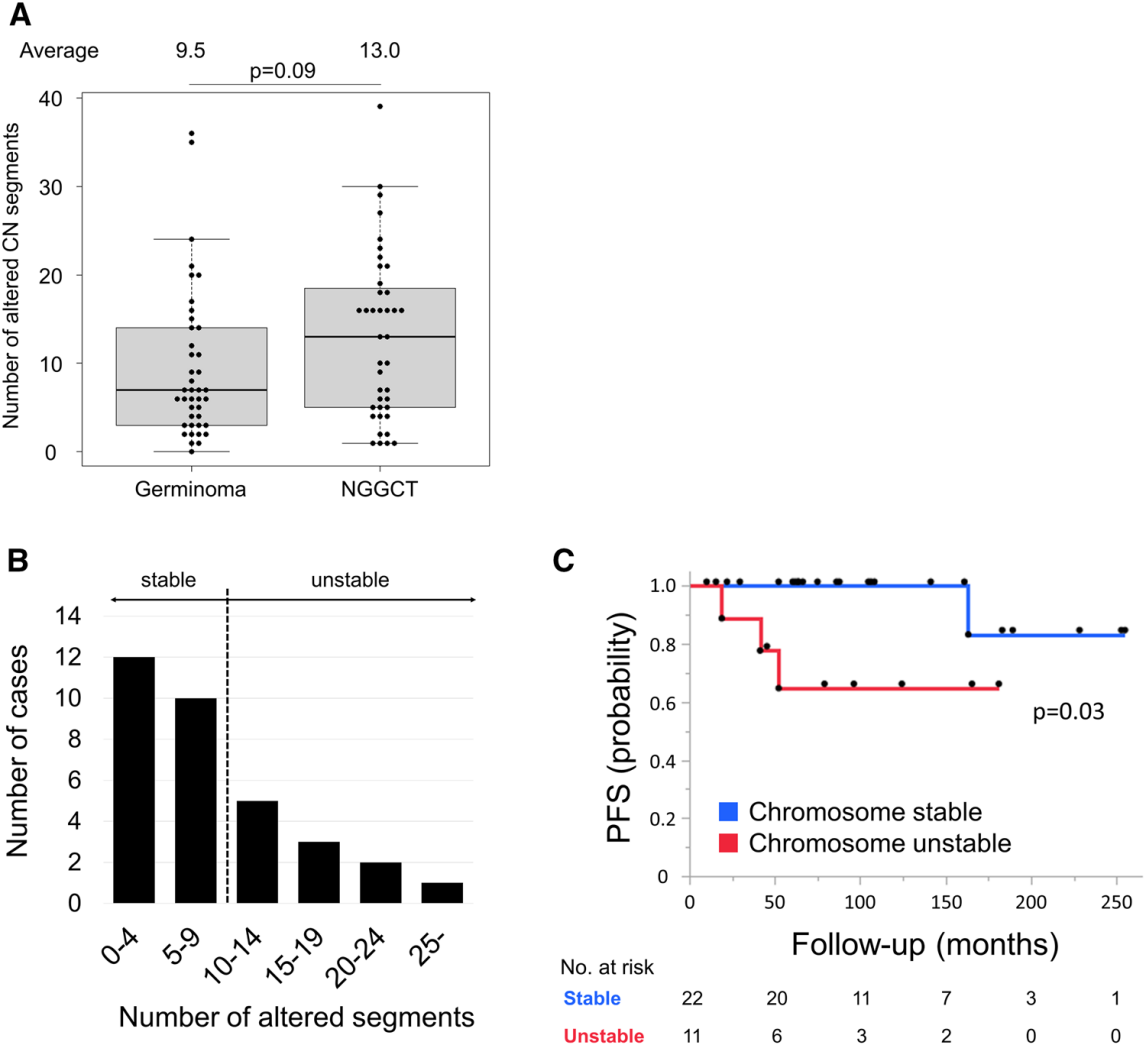
(**A**) Number of altered (gain and loss) segments in copy number analyses of 81 germinoma and non-germinomatous GCT (NGGCT) cases. The number of altered copy number segments tended to be slightly higher in NGGCT cases than in germinoma cases, but the difference was not significant. (**B**) The number of altered copy number segments in germinoma displayed in a histogram. The distribution shows that most cases had a small number of altered segments (< 10, n = 22, stable), although a subgroup showed a high number of alterations (> 10, n = 11, unstable). (**C**) Germinoma cases with a more than 10 altered copy number segments showed shorter progression-free survival (PFS) than other cases.

Next, we interrogated genome-wide copy number changes and investigated whether copy number profile was linked with histopathology and prognosis from a broader perspective. Based on the copy number profiles calculated using the R conumee package under the settings described above, the number of segments across autosomal chromosomes with gains and losses was counted in all 81 cases. The number of altered segments tended to be slightly higher in NGGCT cases compared with germinoma cases, although the difference was not significant (9.5 vs. 13.0 on average, *p* = 0.09) (Fig. 3A). Among primary germinoma cases (n = 33), most cases displayed fewer copy number alterations (number of altered segments < 10, “stable chromosome”, n = 22), while a minority of cases harbored a higher number of alteration (> 10, “unstable chromosome”, n = 11) (Fig. 3B). Germinoma cases with stable chromosomes showed longer PFS than cases with unstable chromosomes (*p* = 0.03) (Fig. 3C). As only one death was recorded among germinoma cases, the association with OS could not be investigated. In addition, no significant correlation was found for prognosis in relation to chromosomal instability in NGGCTs, although similar trends in both PFS and OS were observed (Supplementary Fig. 2A,B).

As germinoma is known to have a variable degree of tumor cell content in their tissue, wherein a subgroup of cases were enriched with a high amount of lymphocytes, a lower log2CN threshold at 0.05 was attempted, and the prognosis was evaluated. As the median number of altered segments using this threshold was 15, germinoma cases were divided into two cohorts, like above. The analysis demonstrated a slightly worse PFS in cases with a higher number of altered segments than those with fewer. However, there was no statistical significance (*p* = 0.13, Supplementary Fig. 3A,B). The fraction of the genome altered (FGA) was computed across the genome^21^; the sum of the coverage of a bin (bases) above or below a log2CN threshold (0.1 or 0.05) was divided by the entire genome. When the threshold of 0.1 was used, the median FGA was 20.2%, and germinoma cases with a higher FGA demonstrated a slightly worse PFS than otherwise, and the difference was not significant (*p* = 0.20, Supplementary Fig. 3C,D). When the 0.05 threshold was used, the median FGA was 48.9%, and again, the PFS was not statistically different (*p* = 0.29, Supplementary Fig. 3E,F).

Tumor cell content was proportional to FGA. The R-squared values in a linear regression model were 0.39 when log2 CN ratio of 0.1 and 0.05 was applied. As a tumor cell content of ≥ 50% was proven to be a poor prognostic indicator in germinoma cases^17^, PFS was assessed using tumor cell content (≥ 50% or < 50%) and the number of altered segments (≥ 10 or < 10) as variables in the Cox regression analysis. This showed that neither of the two factors demonstrated statistical significance, with *p* values 0.21 and 0.09, respectively.

## Discussion

This study aimed to investigate the impact of copy number alterations on histopathology, platinum-resistance and prognosis in CNS GCTs. We found that 3p25.3 gain correlated with the histopathological diagnosis of NGGCT, presence of a YST histological component among NGGCTs, and shorter PFS. However, the finding that 3p25.3 gain was related to chemotherapy-resistance, which would have been represented by the presence of 3p25 gain in recurrent tumor tissues following platinum-based chemotherapy, was not evident in the current CNS GCT cohort.

The hypothesis about the cell of origin for GCTs stands for PGC, and extragonadal GCTs (including CNS and mediastinal GCTs) are hypothesized to originate from mis-migrated PGCs^2,19,20,22–25^. In addition to the morphological similarities between gonadal and extra-gonadal GCTs from histopathological observations, these tumors are reported to be at least partly akin in terms of molecular findings, such as mutational profiles, copy number alterations, methylation status and transcriptomes^26–31^. Under these circumstances, gonadal and extra-gonadal GCTs would fall into the same category of type I or II GCT^1^. In contrast, a previous transcriptome study revealed that CNS and testicular GCTs were clustered differently in a dimensionality reduction method, implying the presence of unique characteristics according to the site of occurrence^3^. Furthermore, the same study comparing CNS and testicular GCTs also showed that copy number alterations were more enhanced in testicular GCTs than CNS GCTs^3^. This study revealed that the 3p25.3 gain may have clinical and histopathological significance in CNS GCTs as in mediastinal and testicular GCTs in part, which may corroborate the existence of shared biological properties between CNS and gonadal GCTs. Nonetheless, the almost equal presence of 3p25.3 gains in primary and recurrent CNS GCTs contrasted with findings in mediastinal and testicular GCTs, where the gain was significantly more prevalent for recurrent tumors. Our results suggest the possibility of site-dependent features of GCTs. Furthermore, the close association between the presence of a YST component as histopathology and 3p25.3 gain suggests that this gain may be a characteristic of Type I GCT, which occurs in neonates and children less than 6 years of age and is histopathologically YST and immature teratoma. The five cases with 3p25.3 gain in the current cohort demonstrated an age range of 2, 16, 16, 19, and 20 years. Although the two cases were diagnosed as high-grade GCTs in the central histopathology review, all the cases had either YST or immature teratoma components in the original histopathology diagnoses in local institutions. The five cases did not match the typical Type I GCT profile, and further investigation is warranted.

In our cohort, 4 of 5 cases with 3p25.3 gain also harbored entire 3p gain. The preceding study of testicular GCTs also showed that many of the samples with 3p25.3 gain appeared to have concomitant 3p gain (11 of 15 per figure)^18^. The study demonstrated the presence of cisplatin resistance in cell lines with 3p25.3; however, further functional study is warranted to prove the significance of the specific region of 3p25.3 gain in tumor biology.

We have previously reported finding 12p gain at a higher frequency in NGGCTs compared with germinoma, particularly in those with a malignant component^15^. Accordingly, the relationship between copy number gain of 3p25.3 and 12p gain was investigated. Among the 20 NGGCT cases with 12p gain, 3 cases showed concomitant 3p25.3 gain. This association was not significant (*p* = 1.0). These two copy number gains thus appear to have independent biological significance. By combining 12p gain and 3p25.3 gains, copy number gains were more closely associated with NGGCT cases, particularly those with malignant components, than each single gain alone.

The present findings also suggested that germinoma cases with a higher number of chromosomal aberrations may manifest shorter PFS (Fig. 3C). One marker-positive case was identified in each of the chromosome-stable and chromosome-unstable groups (GCT91: CSF HCG 58 IU/l and GCT19: serum HCG 61.7 IU/l, respectively), and did not appear to have contributed to prognostic differences. Gain in 12p was more frequent in chromosome-unstable germinoma cases (28.6% vs. 3.7%, *p* = 0.04). No association was found with mutation frequency in the MAPK or PI3K pathways. Germinoma was previously shown to be subdivided into two clusters based on the copy number profile; cases with abundant aberrations across chromosomes, and other cases with few aberrations^31^. Genome-wide hypomethylation including retrotransposons, typically found in germinomas, as well as high expression of embryonic microRNAs, both of which may contribute to genomic instability^1,19,32^. Chromosomal instability, represented as aneuploidy, has been shown to be associated with metastasis, immune evasion and therapeutic resistance, leading to poor prognosis in many cancer types^33,34^. Collectively, germinoma cases with chromosomal instability might constitute a subgroup with poor prognosis warranting more intensive treatment compared to most other cases that are sensitive to standard chemotherapy and radiation therapy. However, using a different log2CN threshold or fraction of the genome altered to divide the cohort, the difference in prognosis was not significant. Furthermore, chromosomal instability appeared to be related to tumor cell content. The small number of recurrences (n = 4) out of the cohort (n = 33) may be one of the limitations of the study; further evaluation using a larger cohort would lead to a more solid analysis.

Some CNS GCTs, mostly NGGCTs and a subset of germinomas, show resistance to standard radiation and chemotherapy^1,18^. These predominantly develop in pediatric and young adult patients, leading to long-term side effects caused by radiation therapy and chemotherapy such as secondary malignancy, intellectual decline, and vascular damage^35–40^. Treatment stratification based on biomarkers is therefore needed. So far, atypical sites of occurrence outside of midline structures (including the neurohypophysis, pineal gland and ventricles), higher tumor cell content in pathological specimens in germinoma, and 12p gain in NGGCTs have been identified as negative prognostic factors^15^. Based on the present results, chromosomal instability in germinoma and 3p25.3 gain in NGGCTs may also be considered as potential markers of poor prognosis. Further in-depth analysis of the combinations of 3p25.3 gain with other chromosomal aberrations in the entire genome would be worthwhile to interrogate potential prognostic markers. Our study was limited by the small size of the cohort, so the findings need to be validated in a larger cohort as a valid biomarker for future treatment stratification. Furthermore, calculation of copy number can be affected by multiple factors, including parameters used in the R package (minfi, conumee) and the samples used as controls. For instance, the previous study analyzing the identical cohort used different parameters, which resulted in slightly different results for cases having 3p25.3 gain (GCT32 and 65). However, the current study aimed to examine the results obtained in testicular GCTs, so the study used the same parameters as those in the testicular GCT study. A more robust method of copy number calculation is warranted for this gain to be valuable in clinical practice in the future.

In conclusion, we propose 3p25.3 gain as a novel marker to predict malignant components and poor prognosis of CNS GCTs. Chromosomal instability in germinoma may identify cases with poor prognosis. In combination with previously reported 12p gain, this may facilitate patient stratification for treatment and prognostication of CNS GCTs. Validation studies in a larger number of patients are underway.

### Supplementary Information

Supplementary Figure 1.

Supplementary Figure 2.

Supplementary Figure 3.

## Data Availability

The datasets generated and/or analyzed during the current study are available from the corresponding author on reasonable request.
