# Supplementary figures and images for: Distinct patterns of copy number alterations may predict poor outcome in central nervous system germ cell tumors
Source: Sci Rep. 2023 Sep 21;13:15760. doi: 10.1038/s41598-023-42842-3 (PMC10514291; doi:10.1038/s41598-023-42842-3)

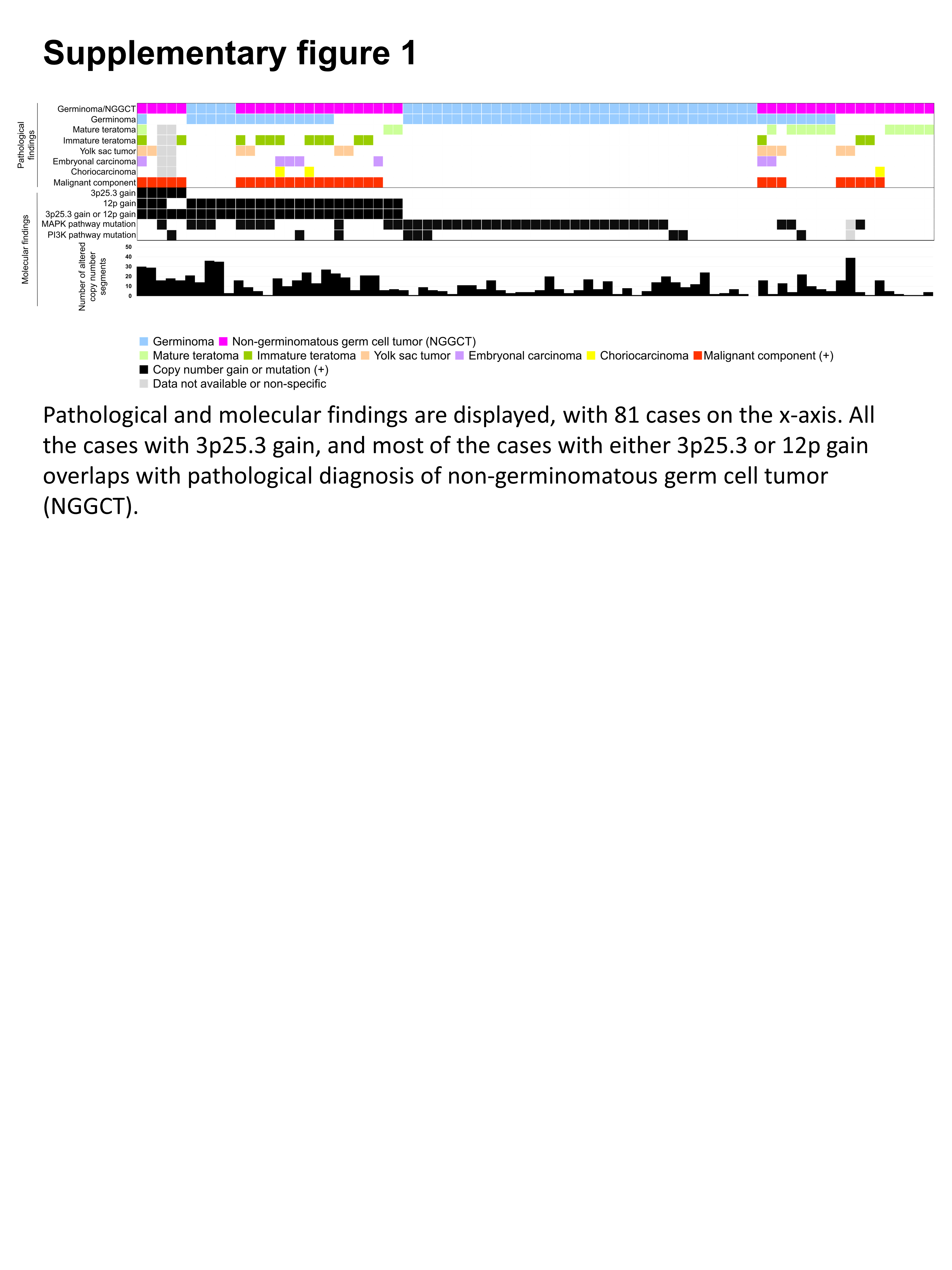

Supplement: Supplementary file 1 — Supplementary Figure 1. [file 41598_2023_42842_MOESM1_ESM.tif]

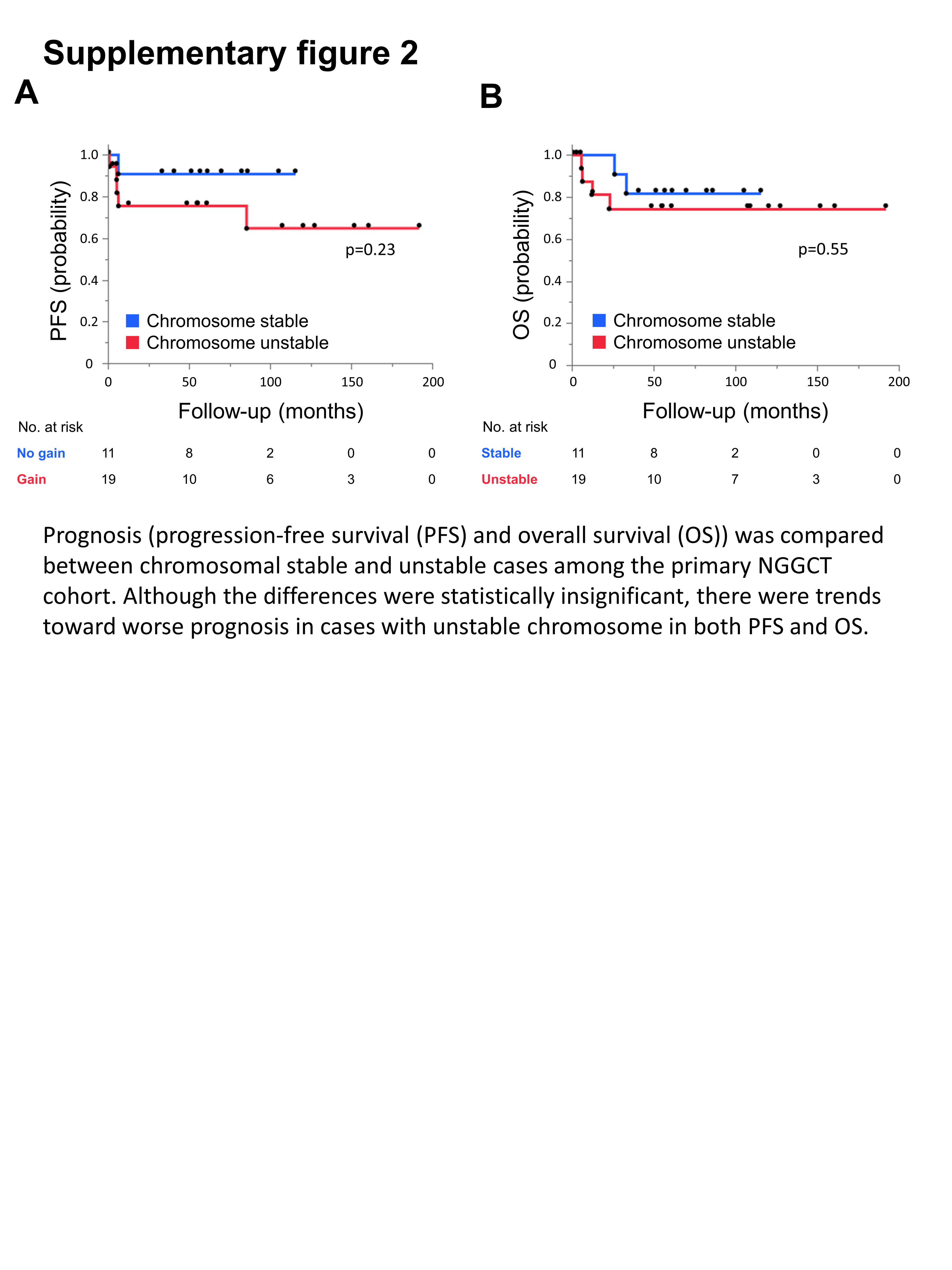

Supplement: Supplementary file 2 — Supplementary Figure 2. [file 41598_2023_42842_MOESM2_ESM.tif]

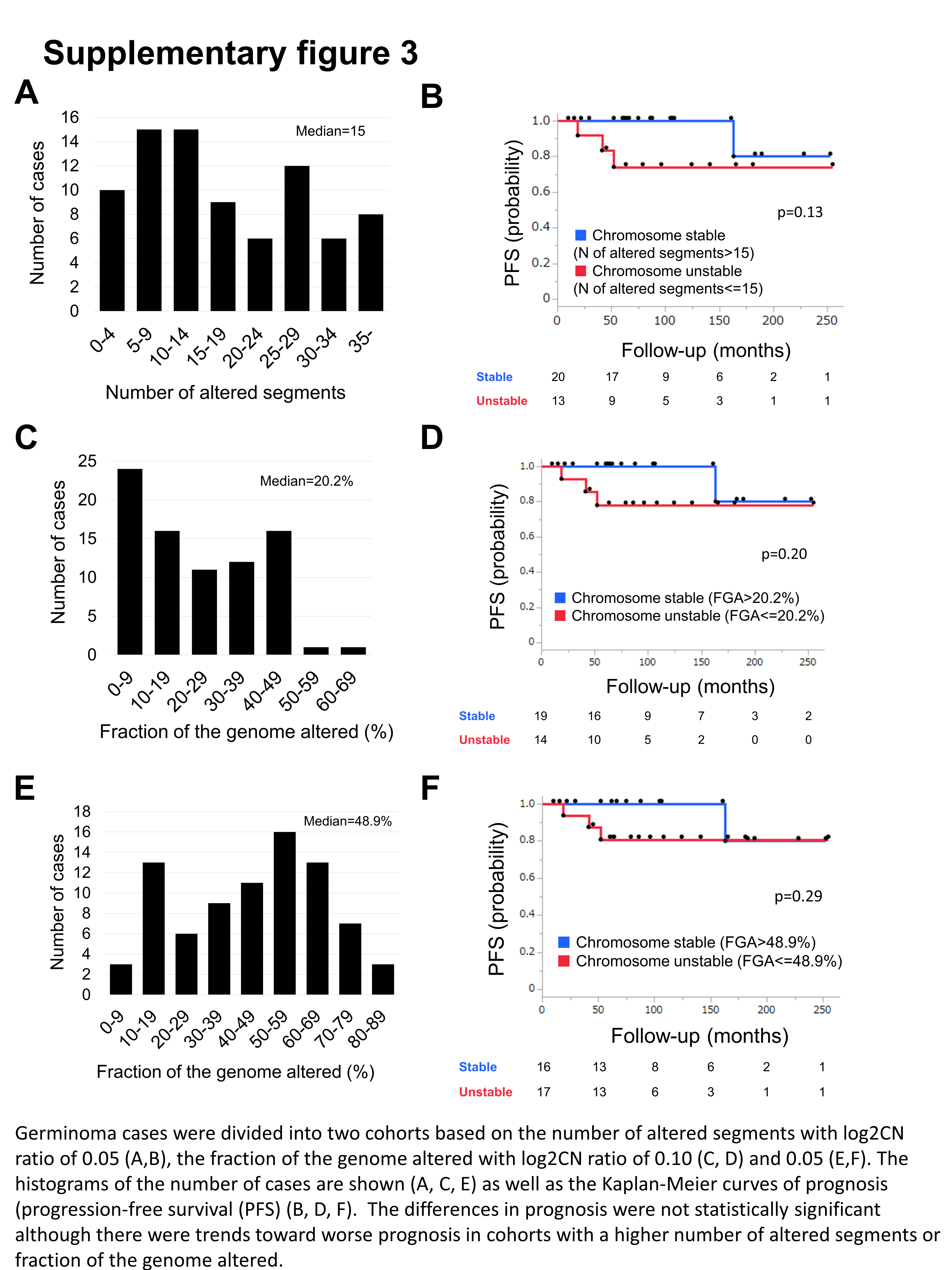

Supplement: Supplementary file 3 — Supplementary Figure 3. [file 41598_2023_42842_MOESM3_ESM.tif]
